# Supplementary material for: Impact of Rearing Duration on Nutritional Composition, Flavor Characteristics, and Physical Properties of Asian Swamp Eel (Monopterus albus)
Source: Foods. 2025 May 9;14(10):1685. doi: 10.3390/foods14101685 (PMC12110856; doi:10.3390/foods14101685)
Supplement: Supplementary file 1 [file foods-14-01685-s001.zip › foods-3620197-supplementary.pdf]

Table S1. Comparative analysis of fatty acid content in *M. albus* muscle from different rearing years.

Fatty Acid	Age 1	Age 3	Age 7	Age 11	Age 22
C10:0 %	0.040±0.000[a]	-	-	-	0.010±0.000[b]
C11:0 %	0.0207±0.006[a]	-	-	-	0.007±0.006[b]
C12:0 %	0.480±0.000[b]	0.697±0.015[a]	0.280±0.000[b]	0.227±0.006[c]	0.230±0.000[bc]
C13:0 %	0.260±0.000	-	0.043±0.006	0.037±0.006	0.050±0.000
C14:0 %	1.983±0.021[a]	2.150±0.046[b]	2.807±0.010[c]	3.113±0.0208[d]	4.083±0.006[e]
C14:1 %	0.193±0.006[a]	0.177±0.006[ab]	0.140±0.000[bc]	0.123±0.006[c]	0.170±0.000[ab]
C15:0 %	1.563±0.006[c]	0.643±0.032[ab]	0.570±0.000[b]	0.523±0.006[a]	0.600±0.000[b]
C16:0 %	22.227±0.040[b]	21.570±0.104[cd]	23.947±0.029[a]	21.853±0.0208[c]	20.990±0.026[d]
C16:1 %	9.247±0.056[a]	11.713±0.065[b]	9.800±0.017[c]	8.247±0.015[d]	8.420±0.010[e]
C17:0 %	1.897±0.076[a]	0.623±0.015[b]	0.643±0.0551[bc]	0.510±0.026[bc]	0.480±0.000[c]
C18:0 %	5.577±0.0153[a]	5.683±0.040[a]	5.230±0.020[b]	4.973±0.0153[c]	4.553±0.006[d]
C18:1n9t %	0.307±0.006	-	-	-	-
C18:1n9c %	18.023±0.015[d]	26.727±0.060[c]	27.037±0.006[b]	28.097±0.045[a]	26.773±0.006[c]
C18:2n6c %	8.877±0.006[d]	17.227±0.090[a]	14.187±0.012[c]	14.950±0.000[b]	16.843±0.015[a]
C20:0 %	0.41±0.010[c]	0.380±0.010[d]	0.443±0.006[b]	0.450±0.000[b]	0.500±0.000[a]
C18:3n6 %	0.197±0.006[a]	-	0.073±0.012[c]	0.137±0.006[b]	0.147±0.015[ab]
C20:1 %	0.447±0.006[e]	0.723±0.015[d]	1.867±0.023[b]	1.900±0.017[a]	1.730±0.010[c]
C18:3n3 %	15.75±0.026[a]	1.440±0.026[e]	1.513±0.015[d]	1.983±0.0153[c]	2.107±0.006[b]
C21:0 %	0.47±0.010[a]	-	0.190±0.000[b]	0.100±0.000[b]	0.097±0.015[b]
C20:2 %	0.827±0.006[a]	0.857±0.025[a]	0.573±0.021[b]	0.517±0.0153[c]	0.553±0.006[b]
C22:0 %	0.983±0.006[a]	0.227±0.025[b]	0.217±0.006[b]	0.217±0.006[b]	0.217±0.006[b]
C20:3n6 %	0.567±0.006[a]	0.370±0.044[abc]	0.220±0.000[bc]	0.160±0.010[b]	0.207±0.006[c]
C22:1n9 %	0.084±0.006[d]	0.200±0.010[c]	0.313±0.015[b]	0.347±0.006[a]	0.320±0.000[b]
C20:3n3 %	1.96±0.020[a]	0.383±0.023[b]	0.287±0.006[e]	0.357±0.006[c]	0.323±0.006[d]
C20:4n6 %	3.843±0.015[a]	3.060±0.066[b]	0.920±0.000[c]	0.653±0.011[e]	0.730±0.010[d]
C22:2n %	0.073±0.006[b]	-	0.127±0.006[d]	0.127±0.006[a]	0.137±0.012[a]
C24:0 %	0.107±0.0153	-	0.057±0.006	0.050±0.000	0.050±0.000
C20:5n3 %	1.420±0.010[c]	0.733±0.031[d]	1.130±0.000[b]	1.660±0.000[b]	1.763±0.006[a]
C24:1 %	-	-	0.260±0.000[b]	0.327±0.006[a]	0.253±0.006[b]
C22:6n3 %	2.170±0.010[e]	4.417±0.057[d]	7.080±0.010[c]	8.367±0.025[a]	7.660±0.010[b]

Note: Value are presented as means ± SD (standard deviation). Superscript letters within the same row represent statistically significant differences ($p < 0.05$).

Table S2. Comparative analysis of hydrolyzed amino acid content in *M. albus* muscle from different rearing years.

Amino Acid	Age 1	Age 3	Age 7	Age 11	Age 22
asp mg/g	0.123±0.005[b]	9.737±0.295[a]	9.808±0.186[a]	8.264±0.216[a]	0.115±0.001[b]
glu mg/g	0.363±0.062[c]	16.902±0.739[a]	15.826±0.295[ab]	14.688±0.635[b]	0.234±0.002[c]
ser mg/g	0.337±0.013[b]	5.823±0.185[a]	5.812±0.328[a]	5.126±0.139[a]	0.322±0.015[c]
his mg/g	3.279±0.012[b]	15.991±0.377[a]	14.122±0.167[abc]	11.674±0.522[ad]	3.359±0.042[cd]
gly mg/g	0.0407±0.001[c]	3.918±0.047[a]	3.698±0.042[ab]	2.912±0.356[b]	0.039±0.001[d]

arg mg/g	0.096±0.000^{c}	10.544±0.120^{a}	10.764±0.524^{a}	8.638±0.412^{a}	0.110±0.002^{b}
ala mg/g	0.372±0.003^{c}	8.134±0.138^{a}	8.186±0.530^{ab}	6.291±0.271^{b}	0.386±0.014^{c}
tyr mg/g	0.127±0.023^{b}	4.834±0.085^{a}	5.561±0.373^{a}	4.289±0.178^{a}	0.088±0.001^{b}
cys-s mg/g	0.070±0.008^{a}	0.477±0.022^{b}	1.089±0.455^{ab}	0.597±0.226^{ab}	0.067±0.000^{a}
val mg/g	0.088±0.014^{c}	6.433±0.131^{a}	6.833±0.309^{ab}	5.367±0.100^{b}	0.088±0.001^{c}
met mg/g	0.014±0.001^{b}	2.713±0.358^{ab}	2.498±0.563^{ab}	1.850±0.130^{a}	0.013±0.000^{b}
phe mg/g	0.113±0.011^{c}	9.056±0.209^{a}	9.441±0.487^{ab}	7.819±0.241^{b}	0.126±0.004^{c}
ile mg/g	0.068±0.009^{d}	7.743±0.160^{b}	8.279±0.514^{a}	6.714±0.135^{c}	0.070±0.002^{d}
leu mg/g	0.091±0.003^{c}	10.551±0.217^{a}	10.664±0.166^{a}	8.857±0.142^{b}	0.089±0.001^{c}
lys mg/g	0.253±0.006^{c}	18.581±0.391^{a}	18.190±0.192^{a}	15.799±0.454^{b}	0.258±0.002^{c}
pro mg/g	0.250±0.015^{c}	8.394±0.335^{a}	7.314±0.122^{a}	5.589±0.505^{b}	0.313±0.025^{c}
TAA mg/g	5.683±0.087^{c}	139.832±2.654^{a}	138.088±4.474^{a}	114.474±3.445^{b}	5.679±0.087^{c}
EAA mg/g	3.906±0.010^{c}	71.069±1.109^{a}	70.028±2.182^{a}	58.080±1.507^{b}	4.003±0.041^{c}
NEAA mg/g	1.777±0.097^{c}	68.762±1.550^{a}	68.060±2.296^{a}	56.394±1.953^{b}	1.676±0.047^{c}
EAA/TAA%	0.687%±0.012%^{b}	0.508%±0.002%^{a}	0.507%±0.001%^{a}	0.507%±0.003%^{a}	0.705%±0.004%^{b}
EAA/NEAA%	2.203%±0.130%^{a}	1.034%±0.008%^{b}	1.029%±0.004%^{b}	1.030%±0.011%^{b}	2.390%±0.044%^{a}

Note: Value are presented as means ± SD (standard deviation). Superscript letters within the same row represent statistically significant differences ($p < 0.05$). TAA: total amino acids; EAA: essential amino acid; NEAA: non-essential amino acid.

Table S3. Comparative analysis of free amino acid content in *M. albus* muscle from different rearing years.

Amino Acid	Age 1	Age 3	Age 7	Age 11	Age 22
asp mg/g	9.000±0.076^{a}	0.070±0.005^{d}	0.112±0.012^{cd}	0.117±0.003^{c}	8.226±0.034^{b}
glu mg/g	9.441±0.818^{a}	0.348±0.036^{b}	0.306±0.008^{b}	0.380±0.024^{b}	8.885±0.022^{a}
asn mg/g	-	0.161±0.019^{b}	0.333±0.004^{a}	0.137±0.040^{b}	-
ser mg/g	4.702±0.171^{a}	0.451±0.014^{b}	0.482±0.021^{b}	0.463±0.023^{b}	4.228±0.010^{a}
gln mg/g	-	1.435±0.035^{c}	2.797±0.021^{a}	1.897±0.030^{b}	-
his mg/g	-	0.251±0.006^{b}	0.169±0.009^{c}	0.292±0.005^{a}	-
gly mg/g	7.910±0.073^{b}	0.032±0.001^{cd}	0.070±0.008^{c}	0.018±0.002^{d}	8.636±0.010^{a}
thr mg/g	7.369±0.098^{a}	0.126±0.003^{c}	0.300±0.044^{c}	0.117±0.004^{c}	5.057±0.024^{b}
arg mg/g	7.042±0.031^{a}	0.117±0.008^{c}	0.084±0.002^{c}	0.085±0.006^{c}	6.482±0.029^{b}
ala mg/g	8.987±0.012^{a}	0.133±0.004^{c}	0.100±0.007^{d}	0.056±0.005^{e}	8.171±0.017^{b}
tau mg/g	0.221±0.012^{e}	0.438±0.007^{b}	0.324±0.004^{c}	0.469±0.0132^{a}	0.238±0.008^{d}
tyr mg/g	3.683±0.019^{a}	-	-	0.050±0.003^{c}	2.951±0.032^{b}
cys-s mg/g	0.414±0.021^{a}	0.070±0.002^{c}	0.081±0.009^{c}	0.071±0.000^{c}	0.218±0.001^{b}
val mg/g	5.071±0.004^{a}	0.036±0.022^{cd}	0.047±0.003^{c}	0.028±0.001^{d}	4.411±0.002^{b}
met mg/g	3.645±0.004^{a}	0.029±0.008^{c}	0.038±0.010^{c}	0.032±0.0123^{c}	2.593±0.012^{b}
phe mg/g	6.802±0.012^{a}	0.069±0.025^{c}	0.067±0.004^{c}	0.073±0.006^{c}	5.781±0.006^{b}

ile mg/g	3.164±0.023^{a}	0.101±0.013^{c}	0.143±0.063^{c}	0.122±0.018^{c}	2.728±0.010^{b}
leu mg/g	9.567±0.019^{a}	0.019±0.007^{c}	0.048±0.036^{c}	0.024±0.003^{c}	7.791±0.013^{b}
lys mg/g	12.964±0.046^{a}	0.067±0.002^{c}	0.073±0.003^{c}	0.063±0.004^{c}	10.566±0.002^{b}
pro mg/g	6.259±0.157^{a}	0.108±0.014^{c}	-	-	5.961±0.003^{b}
TAA mg/g	106.241±1.238^{a}	4.060±0.065^{d}	5.576±0.152^{c}	4.494±0.110^{d}	92.926±0.123^{b}
EAA mg/g	52.265±0.047^{a}	0.697±0.025^{c}	0.886±0.125^{c}	0.800±0.031^{c}	41.880±0.025^{b}
NEAA mg/g	53.976±1.280^{a}	3.363±0.066^{c}	4.690±0.028^{b}	3.69±0.096^{c}	51.046±0.100^{a}
EAA/TAA%	0.492±0.006^{a}	0.172±0.006^{b}	0.159±0.018^{b}	0.178±0.006^{b}	0.451±0.000^{a}
EAA/NEAA %	0.969±0.023^{a}	0.207±0.009^{b}	0.189±0.026^{b}	0.216±0.009^{b}	0.820±0.001^{a}

Note: Value are presented as means ± SD (standard deviation). Superscript letters within the same row represent statistically significant differences ($p < 0.05$). TAA: total amino acids; EAA: essential amino acid; NEAA: non-essential amino acid.
